# Unveiling plant defense arsenal: metabolic strategies in *Brassica oleracea* during black rot disease

**DOI:** 10.1093/hr/uhad204

**Published:** 2023-10-13

**Authors:** Carmen Vega-Álvarez, Pilar Soengas, Thomas Roitsch, Rosaura Abilleira, Pablo Velasco, Marta Francisco

**Affiliations:** Group of Genetics, Breeding and Biochemistry of Brassicas*,*Misión Biológica de Galicia (CSIC), ES-36143, Pontevedra, Spain; Group of Genetics, Breeding and Biochemistry of Brassicas*,*Misión Biológica de Galicia (CSIC), ES-36143, Pontevedra, Spain; Department of Plant and Environmental Sciences, Copenhagen Plant Science Centre, University of Copenhagen, DK-2630, Taastrup, Denmark; Group of Genetics, Breeding and Biochemistry of Brassicas*,*Misión Biológica de Galicia (CSIC), ES-36143, Pontevedra, Spain; Group of Genetics, Breeding and Biochemistry of Brassicas*,*Misión Biológica de Galicia (CSIC), ES-36143, Pontevedra, Spain; Group of Genetics, Breeding and Biochemistry of Brassicas*,*Misión Biológica de Galicia (CSIC), ES-36143, Pontevedra, Spain

## Abstract

Alterations in plant metabolism play a key role in the complex plant–pathogen interactions. However, there is still a lack of knowledge about the connection between changes in primary and specialized metabolism and the plant defense against diseases that impact crops. Thus, we aim to study the metabolic reprograming in *Brassica oleracea* plants upon infection by *Xanthomonas campestris* pv. *campestris* (*Xcc*). To accomplish this, we utilized a combination of untargeted and targeted metabolomics, through UPLC-Q-TOF-MS/MS and ^**1**^H-NMR, in two crop lines differing in resistance that were evaluated at two- and four-week intervals following inoculation (T1 and T2, respectively). Besides, to depict the physiological status of the plant during infection, enzymatic activities related to the carbohydrate pathway and oxidative stress were studied. Our results revealed different temporal dynamics in the responses of the susceptible vs. resistant crops lines. Resistant *B. oleracea* line suppresses carbohydrate metabolism contributing to limit nutrient supplies to the bacterium and prioritizes the induction of defensive compounds such as indolic glucosinolates, salicylic acid, phenylpropanoids and phytoalexins precursors at early infection stages. In contrast, the susceptible line invests in carbohydrate metabolism, including enzymatic activities related to the hexoses turnover, and activates defense signaling related to reactive oxygen species. Thus, each line triggers a different metabolic strategy that will affect how the plant overcomes the disease in terms of resistance and growth. This work provides first insights of a fine-tuned metabolic regulation during *Xcc* infection in *B. oleracea* that will contribute to develop new strategies for plant disease management.

## Introduction

During the past decade, the study of plant metabolome has become increasingly important in understanding the complex interactions between plants and pathogens [1]. Both undergo metabolic changes during infection that perform a critical role in determining the outcome of the disease [1]. When a pathogen invades a plant tissue by overcoming its physical barriers, the plant immune system recognizes it [2] and triggers changes in metabolic pathways [3, 4], including the production of sugars, amino acids, organic acids, flavonoids, alkaloids, terpenoids, and phytohormones, to sign the presence of the pathogen and to activate defense mechanisms [2–4]. In counterpart, pathogens manipulate the plant metabolism to obtain nutrients and create a favorable environment for their growth and survival [5]. All these metabolic alterations comprise a complex metabolic reprogramming, which can have significant consequences on the plant growth, physiology and resistance/susceptibility responses [6–8]. Therefore, the understanding of changes in plant metabolism during infections is crucial to identify disease markers, key metabolic pathways, and metabolites involved in plant immunity, leading to the development of new crop protection strategies.

Metabolomics is then a powerful tool for acquiring a global comprehension of metabolic network regulation. However, the progress of metabolomics research may have been slower compared to other omics technologies like genomics, transcriptomics, and proteomics due to inherent technical limitations in analytical instrumentation and analysis methods [9]. Recently, new advanced data processing systems and the development of several organism-specific metabolomic databases have greatly helped to identify and annotate metabolomic data through structure, mass, fragmentation pattern, or spectral matching [10]. Thus, during the past years plant researchers are beginning to embrace global analyses for enhancing our comprehension of plant immunity at the metabolome level. For instance, the importance of metabolic networks including phytohormones, carbohydrate and secondary specialized metabolism in plant disease interactions is beginning to be established [11–13]. However, the precise impact of these global metabolic alterations on the outcome of plant-pathogen interactions involving crops important for human nutrition remains unclear [14].

**Figure 1:**
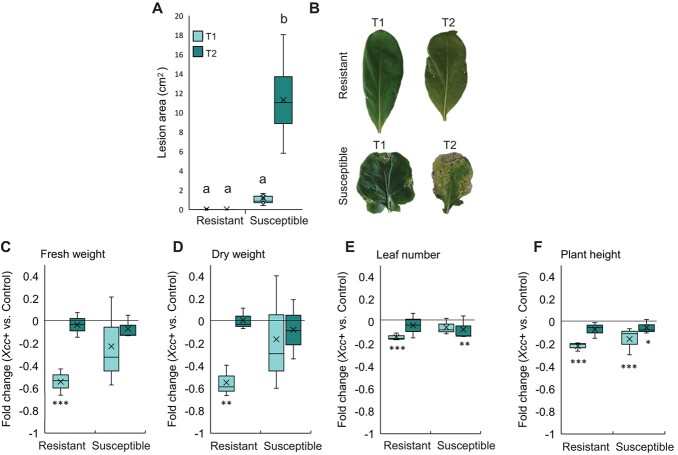
Disease lesions and developmental consequences of black rot disease in resistant and susceptible *B. oleracea* lines. This analysis was conducted at two distinct time points: T1 (two weeks post-infection) and T2 (four weeks post-infection). **A.** Boxplots illustrate the distribution of lesion area on infected leaves. **B.** Appearance of inoculated leaves. The subsequent sections of the figure showcase boxplots presenting fold changes in developmental parameters when comparing *Xcc*-infected plants to control plants, encompassing fresh weight (**C**.), dry weight (**D**.), leaf number (**E**.), and plant height (**F**.). Significant differences were indicated through distinct letters (*P* < 0.05) and asterisks (^*^P < 0.05; ^**^P < 0.005; ^***^P < 0.001). This figure was created using data from a previous study [17], with permission from the authors for reuse and presentation in this new format.

*Brassica oleracea* represents a relevant proportion of the daily food supply of numerous regions worldwide [15]. These crops are severely impacted by the black rot disease, which is triggered by the bacterium *Xanthomonas campestris* pv. *campestris* (Pammel) Dowson (*Xcc*) [16]. We previously found that the infection by *Xcc* causes a loss of weight in young plants of *B. oleracea*; and this growth-immunity tradeoff may be potentially altered by the interaction between primary and the secondary metabolic pathways [17]. Therefore, to offer novel insights into the metabolic processes that occur in *B. oleracea* during infection by *Xcc*, the objective of the present work was to research the metabolic reprograming that plants use to deal with the pathogen in two genotypes: “Badger Inbred-16” previously described as resistant [18] and “Early Big” as susceptible to *Xcc* [19]. This work revealed specific changes in metabolite profiles after inoculation for each crop line that were also dependent upon the time elapsed after inoculation. Metabolic changes in the resistant line, involved the induction of defensive pathways branching off from the shikimate pathway leading to the production of defensive compounds such as phenolics, phytoalexins, and indolic glucosinolates. This upregulation of specialized metabolism was accompanied by a slowdown in primary central metabolism, which regulated by key metabolic enzymes contributed to limit nutrient supplies to the bacterium. In contrast, the susceptible line invests in carbohydrate metabolism to maintain normal growth and develops a different defensive strategy involving the activation of defense signaling related to reactive oxygen species (ROS). This work provides first insights of a fine-tuned metabolic regulation during *Xcc* infection in *B. oleracea* and its contribution to plant immunity.

## Results

### Disease resistance and plant growth

*Xcc*-infection only caused evident lesions in susceptible *B. oleracea* plants (Fig. 1 A,B). According to our previous research [17], resistant line suffers a significant decline in development at two weeks post-infection (T1). One month after the infection (T2) the development traits were very similar in infected and control plants. On contrary, the susceptible line showed a slowdown in plant development at four weeks post-infection, although not as pronounced as in resistant plants (Fig. 1 C–F).

### Non-targeted metabolomic analysis

The cross-validation of PLS-DA analysis indicated a good accuracy, with a goodness-of-fit (R^2^) > 0.99 and high predictability (Q^2^) > 0.60 between treatments (mock-control and *Xcc*-infected samples) for the resistant line at T1 (Table 1 and Supplementary Fig. S1). This indicated that resistance was linked with global metabolomics changes two weeks after inoculation. However, the PLS-DA score plot does not exhibit accurate discrimination models between control and infected samples for the susceptible line at T1 and T2, nor for the resistant line at T2 (Table 1 and Supplementary Fig. S1). Thus, in those samples, the global metabolite profiles did not show any visible effects due to *Xcc* infection. However, we were able to find discernible specific metabolite changes (*P* ≤ 0.05) related to responses during the infection.

**Figure 2:**
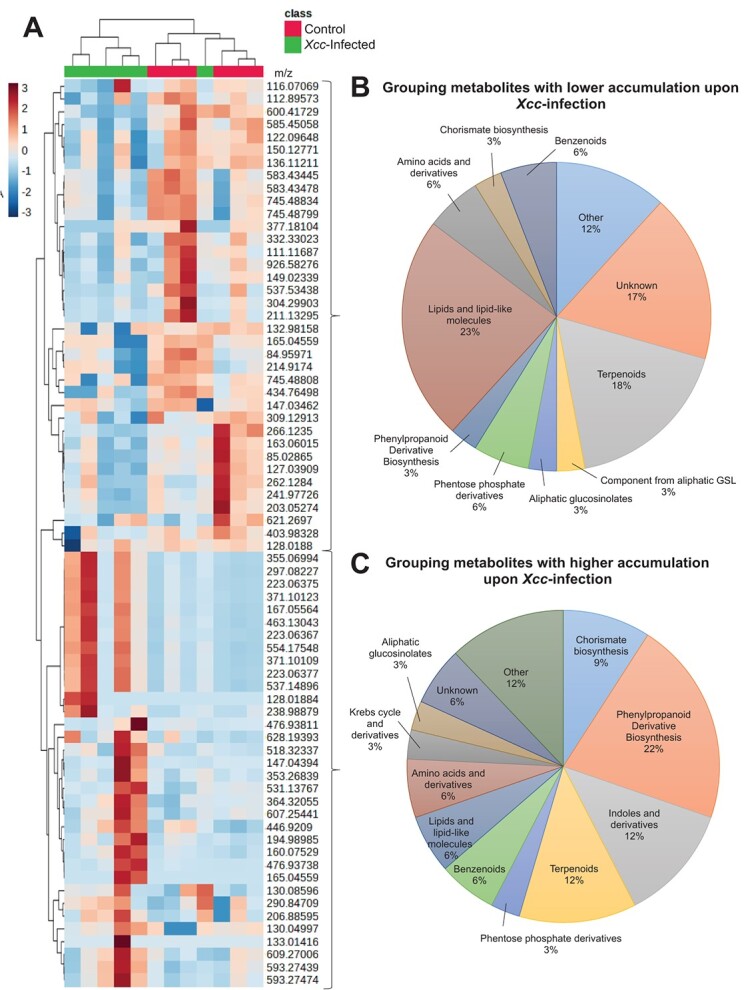
Identification of metabolites implicated in the defense against *Xcc* in the cabbage Badger Inbred-16 at 15 days post-infection. **A.** Clustering heatmap analysis of the intensity of 71 metabolites that exhibited differential accumulation in response to *Xcc* infection. The color code on the scale represents the normalized metabolite abundance, ranging from −3 (intense blue) to 3 (intense red). **B.** Metabolites downregulated during *Xcc* infection. **C.** Metabolites upregulated during *Xcc* infection.

Based on VIP-score > 1.5, 71 metabolites were selected as important features in the PLS-DA analysis on the resistant line at T1 (Supplementary Table S1). Using hierarchical cluster analysis, the 71 selected metabolites were classified into two distinct groups. (Fig. 1 A). One group consists of metabolites with decreased accumulation upon *Xcc*-infection compared with control plants (37 metabolites). Most of these compounds were classified as lipids or lipid-like molecules and terpenoids (Fig. 2 B). The other group comprises metabolites with increased accumulation in infected plants compared with control plants (34 metabolites). Those were metabolites related with phenylpropanoid biosynthesis, indoles and derivatives, terpenoids, and chorismate biosynthesis (Fig. 2 C).

**Table 1:** PLS-DA cross validation details for untargeted metabolomic analysis

**Line and Time**	**Measure**	**1 comps**	**2 comps**	**3 comps**	**4 comps**	**5 comps**
**Resistant T1**	**Accuracy**	0.83333	0.91667	0.91667	**0.91667**	0.91667
	**R2**	0.58819	0.87958	0.94443	**0.99753**	0.99943
	**Q2**	0.34391	0.61958	0.64568	**0.64949**	0.64635
**Resistant T2**	**Accuracy**	0.61538	0.53846	0.46154	0.46154	0.46154
	**R2**	0.52018	0.74113	0.93084	0.95912	0.99791
	**Q2**	−0.053496	−0.56267	−0.78741	−0.89757	−0.79921
**Susceptible T1**	**Accuracy**	0.46154	0.46154	**0.61538**	0.53846	0.61538
	**R2**	0.68162	0.87954	**0.98128**	0.9932	0.9987
	**Q2**	−0.37166	−0.08982	**0.17956**	0.097769	0.17897
**Susceptible T2**	**Accuracy**	0.5	**0.66667**	0.66667	0.58333	0.58333
	**R2**	0.82876	**0.97279**	0.99334	0.99773	0.99924
	**Q2**	0.21599	**0.29497**	0.19791	0.13097	0.20586

**Figure 3:**
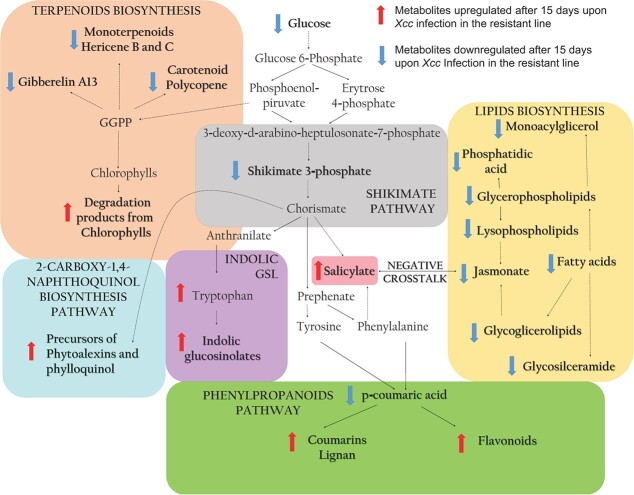
Schematic illustration of the major metabolic pathways that are activated and suppressed during the response to *Xcc* in the resistant line of *B. oleracea*. Metabolites displayed in bold show a significant variation following *Xcc* infection. Indolic GSL, Indolic glucosinolates; GGPP, Geranylgeranyl diphosphate.

Our analysis indicated that most of the upregulated metabolites in *B. oleracea* resistant line during the response to *Xcc* attack were mainly related with downstream pathways from shikimate, which links metabolism of carbohydrates to biosynthesis of secondary specialized compounds. These included intermediates for the biosynthesis of defensive phytoalexins, indolic glucosinolates, salicylic acid, and phenylpropanoids pathways (Fig. 3). Interestingly, while the salicylate biosynthesis was upregulated upon infection, the jasmonate biosynthesis was found to be downregulated (Fig. 3). Besides, chlorophyll breakdown intermediates were also upregulated during infection (Fig. 3).

In concordance with the findings in the resistant line, most of the identified metabolites that were differentially accumulated between treatments in the susceptible line were linked with the phenylpropanoid pathway. Several phenolic compounds significantly decreased after infection, indicating a clear involvement of these secondary metabolites in plant immunity responses (Supplementary Table S2).

### Targeted analysis of the primary metabolism

**Figure 4:**
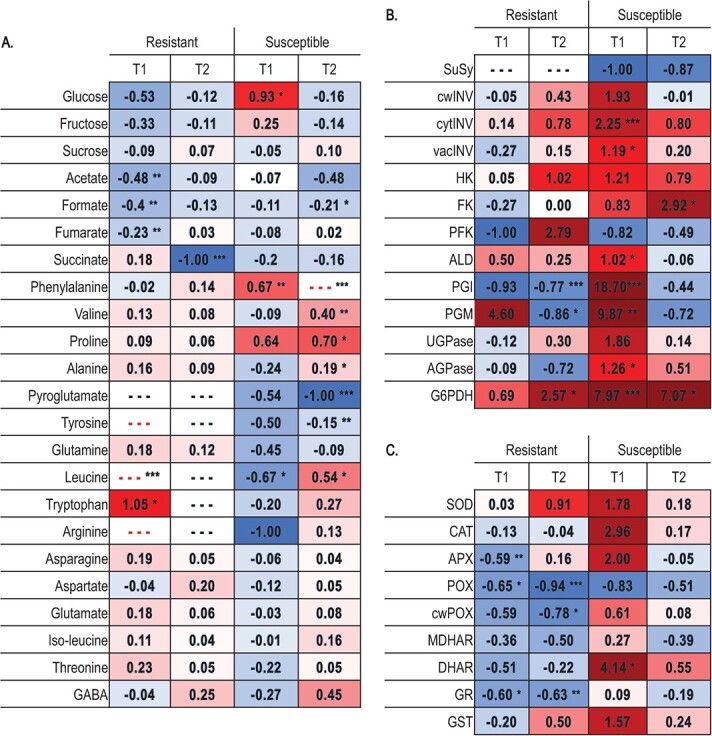
Heatmap displaying **A-** primary metabolites and enzyme activity signatures from **B**- carbohydrate and **C**- ROS scavenging enzymes in resistant and susceptible *Brassica oleracea* lines at T1 and T2. The heatmap in red to blue compares the fold change in each metabolite or enzyme for both lines and times sampled from high to low concentration or relative activity. Fold changes are referred to the variations between *Xcc*-infected to control plants. The asterisks denote statistically significant distinctions between average values based on Student's *t*-test (^*^P < 0.05; ^**^P < 0.01; ^***^P < 0.001). SuSy, sucrose synthases; cwINV, cell wall invertase; cytINV, cytosolic invertase; vacINV, vacuolar invertase; HK, hexokinase; FK, fructokinase; PFK, phosphofructokinase; ALD, aldolase; PGI, phosphoglucose isomerase; PGM, phosphoglucomutase; UGPase, UDP-glucose pyrophosphorylase; AGPase, ADP-glucose pyrophosphorylase; G6PDH, glucose-6-phosphate dehydrogenase; SOD, superoxide dismutase; CAT, catalase; APX, ascorbate peroxidase; POX, peroxidase; cwPOX, cell wall bound-peroxidase; MDHAR, mono-dehydroascorbate reductase; DHAR, dehydroascorbate reductase; GR, glutathione reductase; GST, glutathione S-transferase. “—” appears in black colour when the fold change is not calculable because both values are null, in red colour when control value is null and *Xcc*-infected value is possitive.

**Figure 5:**
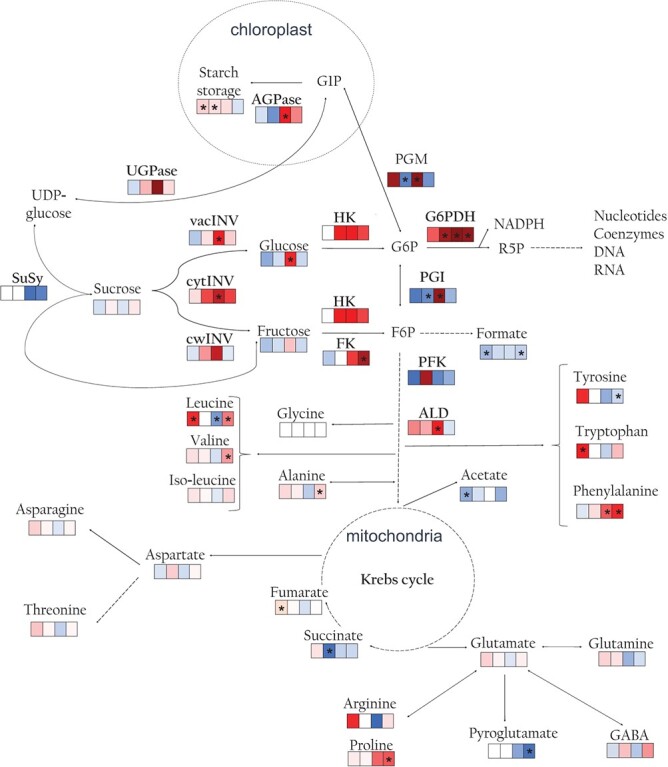
Metabolism reprogramming overview in resistant and susceptible *B. oleracea* lines at 15 and 30 days (T1 ans T2) upon *Xcc* race 1 infection. For each metabolite measured, there is a table made up of 4 squares and from left to right they refer to: Resistant line at T1; Resistant line at T2; Susceptible line at T1; Susceptible line at T2. The colours of the primary metabolites and enzymes in this figure depend on the fold changes from *Xcc*-infected to mock control (View heatmap from Fig. 4). Red to blue from high to low fold change. In cases when is not posible to calculate the fold change because the concentration or activity is not detectable in the control plants and detectable in *Xcc* infected plants the dark red color was chosen. Statistically significant differences between treatments (control and *Xcc* infection) are marked with an asterisk. ¡The information about starch proceed to literature. G6P, Glucose 6-phosphate; G1P, Glucose 1-phosphate; R5P, Ribose 5-phosphate; SuSy, sucrose synthase; cwINV, invertase from the cell wall; cytINV,cytosolic invertase; vacINV, vacuolar invertase; HK, hexokinase; FK, fructokinase; PFK, phosphofructokinase; ALD, aldolase; PGI, phosphoglucose isomerase; PGM, phosphoglucomutase; UGPase, UDP-glucose pyrophosphorylase; AGPase, ADP-glucose pyrophosphorylase; G6PDH, glucose-6-phosphate dehydrogenase.

The concentration of glucose was higher in control than inoculated plants in the resistant line, especially at T1 (Supplementary Table S1). The susceptible line followed the opposite trend at T1, since glucose was significantly higher in the inoculated plants compared to the control ones (Fig. 4 A, Fig. 5, Supplementary Fig. S2 A). No significant differences were found in fructose and sucrose concentrations, although changes in fructose showed a similar trend than glucose (Fig. 4 A, Fig. 5, Supplementary Fig. S2 A).

Acetate, formate and fumarate concentration significantly decreased at T1 during the infection in the resistant line, while the concentration of succinate decreased significantly at T2. In the susceptible line, the formate concentration decreased significantly with the inoculation at T2 (Fig. 4 A, Fig. 5, Supplementary Fig. S2 B).

Regarding amino acids, in the resistant line leucine and tryptophan increased significantly with the infection at T1. In the susceptible line, phenylalanine increased with the infection in the susceptible line at both times, while valine, proline, and alanine increased in the inoculated plants respect to control at T2. Pyroglutamine and tyrosine decreased with the infection at T2. Leucine decreased in *Xcc*-infected respect to control plants at T1, while the opposite trend was observed at T2 (Fig. 4 A, Fig. 5, Supplementary Fig. S2 C).

### Changes in enzymatic activity

The results evidenced significant changes in enzymatic activities related to carbohidrate metabolims (C-enzymes) in both lines upon infection. In the resistant line, the effects of the *Xcc* attack on these enzymatic activities were evident at T2, when PGI and PGM activities decreased significantly in *Xcc*-infected compared to control plants, and the G6PDH activity increased. With respect to the susceptible line, the enzymatic activities cytINV, vacINV, ALD, PGI, PGM, AGPase increased with the inoculation at T1, FK activity at T2 and G6PDH activity at both times (Fig. 4 B, Fig. 5, Supplementary Fig. S3). Regarding antioxidant enzymes (A-enzymes) our results showed that APX activity decreased with the infection in the resistant line at T1. The activities of POX and GR decreased significantly with the infection at both times, while cwPOX activity decreased only at T2. On the contrary, in the susceptible line, the DHAR activity had a strong and statistically significant increase during the infection at T1 (Fig. 4 C, Supplementary Fig. S4).

## Discussion

Alterations in the plant metabolism are crucial for determining the most efficient use of available resources when confronted with various environmental stresses [1]. The present study revealed the importance of a fine-tuned metabolic regulation throughout plant-pathogen interactions and its impact in plant defense. Changes in metabolite profiles and enzymatic activity after inoculation were specific to each crop line (resistant and susceptible); and were also dependent upon the time after infection. Thus, plants must timely coordinate conflicting demands when exposed to the pathogen for a better outcome of the infection.

Although the disease damages were much more noticeable in the susceptible line (Fig. 1 A), development slowed down, specially in the resistant line upon *Xcc* infection al T1 (after this line recovered) (Fig. 1 C-F). Primary metabolic reprogramming was determinant for the defensive state of the resistant line. At two weeks after infection, this line decreased glucose content (Supplementary Table S1) and TCA intermediates (Fig. 4 A, Fig. 5, Supplementary Fig. S2B) and slowed down carbohydrate metabolism (Fig. 4 A, Supplementary Fig. S2, Supplementary Table S1). The drop in chlorophyll production and the increase in their breakdown products (Supplementary Table S1) also suggested a down-regulation of photosynthesis. Carbohydrate metabolism plays important roles in many physiological mechanisms and sugar efflux is an important player in plant diseases [20]. To gain insights on how this drop of sugars is produced, we measured the activities of enzymes related to the turnover of hexoses. Interestingly, the resistant line exhibits a decrease in PGI and PGM activities during *Xcc* attack (Fig. 4 B, Fig. 5, Supplementary Fig. S3), which may limit the flux of carbohydrates through central metabolism since these enzymes are involved in glycolysis. This phenomenon would divert the nutrients towards the pentose phosphate pathway (through the action of the enzyme G6PDH) (Fig. 4 B, Fig. 5, Supplementary Fig. S3) and the shikimate pathway (from alternative steps to the glycolysis pathway) (Fig. 3, Supplementary Table S1) leading to the amino acids biosynthesis among other defensive compounds [21]. These pathways are implicated in the production of defense compounds, cell wall reinforcement, the oxidative stress scavenging and the activation of the plant's immune response [21, 22]. This work supports the idea that there is a coordination of defence responses with primary carbohydrate metabolism and assimilate partitioning, possibly related with an induction of sink and a repression of source activities [23–25].

Global metabolomic changes after infection were not significant in the susceptible line. However, specific changes in several metabolites were associated with the response to the pathogen. At initial stages of infection, this line increased glucose content (Fig. 4 A, Fig. 5, Supplementary Fig. S2 A). This was in harmony with an increased activity of several enzymes related to carbohydrate metabolism involved in the synthesis and degradation of carbohydrates, like the glycolysis pathway (PGI, PGM, FK and ALD), the conversion of sucrose to glucose (cytINV and vacINV), the starch biosyntesis (AGPase) and the redirection of resources to the pentose phosphate pathway (G6PDH) (Fig. 4 B, Fig. 5, Supplementary Fig. S3). The upregulation of primary metabolism in the susceptible line ensures enough energy levels for plant growth and defense responses or even can result from the pathogen modifying the plant's metabolism to meets its own energy requirements [5]. The strategy consistent in upregulating the primary metabolism also modulates defense responses [26] such as ROS production [27] and ROS scavenging enzymes [28], which are influenced by glucose availability. The pentose phosphate pathway is an important part of glucose metabolism, to produce NADPH, the major cofactor of those antioxidant enzymes [29]. Later in the infection, the initial glucose increment could be partially reallocated into products of the pentose phosphate pathway and other defensive pathways [30].

One notable aspect of the *B. oleracea* infection caused by *Xcc* was the substantial time-dependent reprogramming of secondary metabolism (Fig. 2, Fig. 3, Supplementary Fig. S1, Supplementary Table S1). Infection in the resistant line triggered the activation of plant secondary biosynthetic pathways, mainly phenylpropanoids, but also indolic glucosinolates, phytohormones and phytoalexins precursors. In contrast, decreased content of phenylpropanoid derivatives were found in the susceptible line after infection (Supplementary Table S2), suggesting a key role of this class of compounds in the defense response of *B. oleracea* against *Xcc*.

The phenylpropanoid pathway plays a crucial role in the synthesis of various compounds, including lignin, and serves as a precursor for the production of several other important compounds such as flavonoids, coumarins, hydroxycinnamic acid conjugates, and lignans [31]. Hydroxycinnamic acids are important precursors of lignin biosynthesis and exhibit an increase in *Brassica* plants after infection. Among this king of compounds, *p*-coumaric acid and their conjugates were overrepresented in the defense response (Fig. 3, Supplementary Table S1). They provide structural support and resistance by inhibiting pathogen dissemination and reinforcing the secondary cell walls of plants [32]. Several flavonoids were also accumulated in plant leaves after the infection in the resistant line (Fig. 3, Supplementary Table S1). Induced resistance mediated by flavonoids against biotic stress is beginning to be elucidated by the potential toxicity of these metabolites to suppress the pathogenicity of invaders and/or to relieve stress-related oxidative pressure [33]. Further research is needed to understand the molecular mechanisms that regulates phenolics induction during plant-pathogen interactions.

The significance of plant hormones is well-established in the context of plant disease interactions, where specific hormone signatures are associated with attack by biotrophic or necrotrophic pathogens [34]. According to previous research [17], our data supports that the salicylic acid is a master regulator of plant resistance to *Xcc* in *B. oleracea* resistant line. On the contrary, jasmonic acid was downregulated after infection. These findings are consistent with the fact that *Xcc* acts as biotrophic in the resistant line [35]. Together with jasmonic acid, a multitude of lipids related to the synthesis of this hormone decrease. It suggests a repression in jasmonic acid biosynthesis by the salicylic acid [36].

Glucosinolates are integral components of the immune system in *Brassicaceae* plants regulated by phytohormone signaling [37]. These compounds are synthesized and stored constitutively as phytoanticipins. However, in response to pathogens, de novo synthesis can be triggered to disrupt the infection [38–40]. In agreement with previous reports, *Brassica* resistance to *Xcc* was mediated by the induction of indolic glucosinolates [17, 38]. These compounds are normally found in relatively low concentration in leaves, but pathogens redirect glucobrassicin biosynthesis to 1/4-hydroxy and 1/4-methoxy substituted indolic glucosinolates [41]. Along with glucobrassicin (Supplementary Table S1), our global metabolomics analysis found highly increased levels of 1-methoxy-indol-3-yl-methyl glucosinolate (neoglucobrassicin) in the resistant line (Supplementary Table S1). Reactions leading this kind of glucosinolates are catalysed by members of the subfamily CYP81F of cytochrome P450s and has been proposed to be regulated by salicylic acid [42]. Products of these compounds such as indole-3-carbinol (I3C) and/or indole-3-acetonitrile (IAN), the camalexin precursor are required for pathogen resistance [43].

The oxidative pentose phosphate pathway is a major source of reducing power (i.e., NADPH production), provides metabolic intermediates for biosynthetic processes (i.e., fatty-acid synthesis) and sustains the required redox potential to safeguard against oxidative stress [44, 45]. Its main regulatory step is catalyzed by G6PDH [46], which is involved in plant immunity because forms an immune signaling module downstream of pattern recognition receptors, establishing a connection between protein phosphorylation cascades [45]. Recent years have witnessed an interest of this pathway on plant-pathogen interactions. Our research on this pathway showed that both *Brassica* lines (resistant and susceptible) increased G6PDH (Fig. 4 B, Fig. 5, Supplementary Fig. S3), which would contribute to adjust the NADPH/NADP ratio during the pathogen attack. NADPH participates in the turnover of glutathione, employed by cells to reduce reactive oxygen species (ROS). Coincidently, the activity of the antioxidant enzyme DHAR increases in the susceptible line during the first stages of infection (Fig. 4 C, Supplementary Fig. S4). DHAR reduces dehydroascorbate to ascorbate using reduced glutathione as an electron donor [47]. Contradictorily, the activity of several antioxidant enzymes decreases at T1 in the resistant line, suggesting that oxidative stress was already overcome in this line at the time that we did the measurements.

This work provides new insights into the metabolic processes that occur in *B. oleracea* during infection caused by *Xcc* and its contribution to plant immunity*.* Each line, resistant and susceptible, triggers a different metabolic strategy that will affect the capability of the plant to restrict the growth and/or development of the pathogen. The downregulation of primary metabolism in the resistant line of *B. oleracea* may serve to limit nutrient availability for bacterial growth and to preserve metabolic resources for accumulation of specialized compounds involved in plant defense. This strategy explains the growth-immunity tradeoff detected in the resistant line during the early stages of infection in a previous work. In contrast, it seems that the susceptible line upregulates primary metabolism during the initial stages of the infection, possibly to support the plant's normal growth while accommodating the initial energy demand, or maybe the mentioned upregulation is just a consequence of the pathogen manipulating the plant's metabolism to satisfy its own requirements. This strategy does not interrupt the sugar supply to the pathogen and important chlorotic and necrotic lesions appear but activate another defense signaling such as those related to ROS. Both upregulation and downregulation of primary metabolism have been reported in different pathosystems previously. Nevertheless, in the *B. oleracea*-*Xcc* interaction, the downregulation of primary metabolism appears to be the more promising defensive strategy to successfully overcome the infection. New studies on this complex and multi-layer defense response will help to understand the fine-tuned metabolic regulation that take place during plant-pathogen interactions and develop new strategies for plant disease management in *Brassica* crops.

## Material and methods

### Plant growing and design of experiments

Seeds of two doubled haploid lines of *B. oleracea* with different responses to *Xcc* race 1 were employed: the resistant cabbage Badger Inbred-16, and the susceptible line derived from broccoli (referred to as "Early Big") [17], were grown in pots with 2.5 L of peat (Gramoflor GmbH and Co. KG Produktion in Vechta, Germany). The plants were cultivated in a greenhouse under controlled conditions with an 8-hour photoperiod and an average day/night temperature of 20/15°C. The experimental design consisted on split-plot arrangement, with the main plots comprising control plants and plants inoculated with *Xcc* race 1. At six weeks after sowing, we performed the inoculation with *Xc*c race 1, in the second youngest leaf of each plant (from the apex). Inoculation involved the injection of a bacterial suspension (5 × 10^8^ cfu mL^−1^) into three points on each leaf using sterilized forceps wrapped in cotton [38], while control plants were mock-inoculated. Two equivalent sets of plants were arranged within each plot to collect samples at two- and four-week intervals after inoculation (referred to as T1 and T2). Each condition consisted of three repetitions, with five plants per repetition, randomly assigned within the experiment.

### Sample collection

A section measuring 1 cm^2^ was excised from each leaf and promptly frozen in liquid nitrogen. Subsequently, the pieces that constitute each repetition were combined and mixed together to create bulk samples for enzymatic assays*.* Finally, each plant was individually cut and stored at −80°C. Subsequently, the plants were lyophilized for 72 hours using a BETA 2–8 LD plus lyophilizer (Christ GmbH, Osterode am Harz, Germany). The dried material was powdered with an IKA-A10 mill (IKA-Werke GmbH & Co.KG). Finally, the dry material was used to perform untargeted metabolomic analysis and to analyze the primary metabolites.

### Untargeted metabolomic analysis

This analysis was carried out through non-targeted approach based on LC-QTOF instruments coupled to MS/MS fragmentation patterns and UV spectra. Basically we used the procedure described by [48, 49], performing two successive extractions with 20 mg of lyophilized powder. The extractions were done in 80% aqueous methanol, resulting 2 mL of supernatant, which was filtered with 0.22-micrometer microporous PTTE filter and diluted at 20% in volume in 80% aqueous methanol. Six samples from each condition from three different replicates were analyzed. For metabolomic composition analysis, a volume of five microliters from each sample was injected into an ultra-high-performance liquid chromatography (UHPLC) system (Thermo Dionex Ultimate 3000 LC) paired with a quadrupole time-of-flight mass spectrometer (UPLC-Q-TOF-MS/MS) (Bruker Compact™) equipped with a heated electrospray ionization (ESI) source. The chromatographic separation was carried out using an Intensity Solo 2 C18 column (2.1 × 100 mm 1.7 μm pore size, Bruker Daltonics, Germany) by utilizing a binary gradient solvent mode. The separation was achieved employing 0.1% formic acid in water (solvent A) and acetonitrile (solvent B). The following gradient was employed: From 0 to 4 minutes, the solvent contained 3% of component B. Subsequently, the percentage of component B increased from 3% to 25% between 4 and 16 minutes. Following that, the percentage continued to rise from 25% to 80% during the interval of 16 to 25 minutes. At 25 to 30 minutes, there was a further increase in the percentage of component B from 80% to 100%, which was maintained until 32 minutes. Afterward, the percentage of component B decreased from 100% to 3% between 32 and 33 minutes, and this value was held steady until 36 minutes at 3% B. A flow rate of 0.3 mL/min was set, and the column temperature was maintained at 35°C. Mass spectrometry (MS) data were taken with an acquisition rate of 2 Hz across a mass range of 50–1200 m/z. Both positive and negative polarities (±) of the electrospray ionization (ESI) mode were employed, utilizing specific conditions: a gas flow of 8 L/min, nebulizer pressure of 38 psi, dry gas flow of 9 L/min, and a dry temperature of 220°C. Capillary and end plate offset were set to 4500 and 500 V, respectively. The stability of the LC-QTOF system was tested by injecting chloramphenicol three consecutive times (ESI – mode; **Δ**RT < 0.02 min; **Δ**m/z = < 0.003) and subsequently triphenyl phosphate (ESI + mode; **Δ**RT < 0.02 min; **Δ**m/z < 0.003). MS/MS analysis was conducted on pooled samples for each day and condition. Ions were targeted based on accurate mass and retention time (RT) data obtained earlier, and fragmented of ions by employing varying collision energy ramps from 15 to 50 eV.

Finally, the raw data from the UHPLC-QTOF were uploaded and processed by the commercial software MetaboScape 4.0 (Bruker Daltoniks, Germany) following [50]. The peak detection and data alignment were proceeded automatically using the T-ReX 3D algorithm. The peak results obtained from MetaboScape 4.0 software were imported into MetaboAnalyst [51] for statistical analysis. To eliminate non-informative variables, the data were filtered using the interquantile range filter (IQR). Additionally, Pareto variance scaling was carried out to normalize the data and to eliminate the offsets and equalize the relevance of ions with high and low abundance levels [52]. The resulting three-dimensional matrix (consisting of peak indices, samples, and variables) underwent multivariate statistical analysis to determine how robust is the response to *Xcc* in each comparison, with the supervised partial least squares discriminate analysis (PLS-DA) methodology. PLS-DA models were cross-validated using Q^2^ and R^2^ parameters. The relevance of each metabolite in PLS-DA models was quantified using the variable importance in projection (VIP) score from the fist principal component. Based on VIP > 1.5, metabolites associated with the response to the infection were distinguished. Features altered due to the infection were selected for tentative metabolite identification.

The tentative metabolite identification was performed using accurate metabolite masses reported in different publicly available databases, such as KEGG (https://www.genome.jp/kegg/), PubChem (https://pubchem.ncbi.nlm.nih.gov/) and HMDB (https://hmdb.ca/). All databases were accessed between January 1st 2023 and February 6th 2023. Additionally, MetaboScape 4.0 and SIRIUS 4 [53] were used for further partial identification of the most significant metabolites by comparing the MS/MS fragmentation patterns with reference compounds listed in the aforementioned databases and the available literature.

### Targeted metabolomic analysis

Primary metabolites were analyzed with proton nuclear magnetic resonance spectroscopy (^1^H-NMR) in the metabolomics platform from CEBAS-CSIC, as previously described [54]. Around 50 mg of the dry material were used for estimating the concentration of hexoses and sucrose, organic acids and amino acids. Following this procedure, we identified and quantified two hexoses: glucose and fructose; one disaccharide: sucrose; four organic acids: acetate, formate, fumarate, succinate; sixteen amino acids: Phenylalanine, valine, proline, alanine, pyroglutamate, tyrosine, glutamine, leucine, tryptophan, arginine, asparagine, aspartate, glutamate, iso-leucine, threonine, and GABA. This procedure was carried out with six randomly selected individual plants from each condition, from three different repetitions.

The GLM procedure of SAS was utilized to conduct analyses of variance [55]. Fixed factors included lines, harvest time, and treatments, while replications were treated as a random factor. ANOVAs were conducted separately for each time point and line. Mean comparisons between treatments were performed using a Student's *t*-test at a significance level of *P* ≤ 0.05. The statistical analyses were done separately by lineand time.

### Extraction of total proteins and enzyme activity profiling

Protein extraction was done as described by Jammer and collaborators [56] to obtain the intracellular and the cell wall extracts. The harvested material frozen in dry ice was ground, weighed and subjected to homogenization in a cold extraction buffer prepared as described by [57]. After 30 min of strong shaking at 4°C, the samples were centrifuged during 10 min at 4°C at 13500 g to separate the supernatant and the pellet. The supernatant was dialysed overnight against 20 mM phosphate buffer pH 7.4 to obtain the intracellular extract. During the dialysis process the buffer was renewed at least three times. The pellet of the extraction was washed three times in cool water and 1 mL of high salt buffer [57] was added to mix by shaking overnight at 4°C. Then the samples were centrifuged at 4°C during 10 min at 10000 g. This resulting supernatant obtained from the extraction pellet was dialysed as mentioned above to get the cell wall extract. Extracts were stored at −20°C for measuring the enzyme activity.

Subsequently, 2–4 μL of the corresponding protein extract were employed to determine the enzyme activities signatures of several key enzymes from the carbohydrate and oxidative stress pathways in 96-well microtiter plates (Sarstedt, Nümbrecht, Germany). Carbohydrate enzyme activities (C-Enzymes) were estimated according to the method of Jammer and collaborators [56]. We estimated the activities of sucrose synthases (SuSy), invertases from the cell wall (cwINV), the cytosol (cytINV) and the vacuoles (vacINV), hexokinase (HK), fructokinase (FK), phosphofructokinase (PFK), aldolase (ALD), phosphoglucose isomerase (PGI), phosphoglucomutase (PGM), UDP-glucose pyrophosphorylase (UGPase), ADP-glucose pyrophosphorylase (AGPase), and glucose-6-phosphate dehydrogenase (G6PDH). The intracellular extract was used to determine the activity of all the C-Enzymes except cell wall invertases. We employed the method of [58] to estimate the following antioxidant enzyme activities (A-Enzymes): superoxide dismutase (SOD), catalase (CAT), ascorbate peroxidase (APX), peroxidase (POX), cell wall bound-peroxidase (cwPOX), mono-dehydroascorbate reductase (MDHAR), dehydroascorbate reductase (DHAR), glutathione reductase (GR), and glutathione S-transferase (GST). The intracellular extract was employed to determine the enzymatic activity of all the A-Enzymes, except cwPOX. Enzymatic activities were normalized by fresh weight. Data transformation and statistical analysis were performed as described in targeted metabolomics analysis section.

## Acknowledgements

This research was supported by the research projects PID2021-126472OB-I00 and RTI2018-094650-J-100 of the Ministry of Science and Innovation, the Government of Spain. Carmen Vega-Álvarez acknowledges a PFI fellowship from the Spanish Ministry of Science and Innovation. Marta Francisco acknowledges the Ramón y Cajal Research Program (RYC2019-027834-I) through the MCIN/AEI/10.13039/501100011033 and “ESF Investing in your future”. Thomas Roitsch would like to acknowledge funding by the Ministry of Education, Youth and Sports of Czech Republic within the National Sustainability Programme I (NPU I), grant number LO1415. The authors want to thank Juan Carlos Fernández, Victor Rodriguez, Rogelio Santiago, and Ana Carballeda at MBG and to Mohsina Ferdous and Chandana Pandey at the University of Copenhagen.

## Author Contributions

M.F. and P.S. conceived and planned the experiments. C.V.A., M.F., and P.S. carried out the plant trials setup analyzed the data and wrote the manuscript. C.V.A. and R.A. carried out the laboratory work. T.R. supervised the enzymatic assays. P.V. contributed to the untargeted metabolomic analysis. All authors read and improved the final manuscript.

## Data availability

Metabolomics data have been archived in the database EMBL-EBI MetaboLights (doi:10.1093/nar/gks1004; PubMed PMID: 23109552) with the identifier MTBLS7815.

The other data will be available upon a reasonable request.

## Conflict of interest statement

The authors declare no conflict of interest.

## Supplementary Data

Supplementary data is available at *Horticulture Research* online.

## Supplementary Material

Web_Material_uhad204
